# *Agaricus brasiliensis* KA21 May Prevent Diet-Induced Nash Through Its Antioxidant, Anti-Inflammatory, and Anti-Fibrotic Activities in the Liver

**DOI:** 10.3390/foods8110546

**Published:** 2019-11-04

**Authors:** Anna Nakamura, Qi Zhu, Yoko Yokoyama, Naho Kitamura, Sena Uchida, Kayo Kumadaki, Kazuo Tsubota, Mitsuhiro Watanabe

**Affiliations:** 1 Systems Biology Program, Graduate School of Media and Governance, Keio University, Kanagawa 252-0882, Japan; anna87@sfc.keio.ac.jp (A.N.); yyokoyama-kyt@umin.ac.jp (Y.Y.); nahoshi@sfc.keio.ac.jp (N.K.); s.uchida.1963@keio.jp (S.U.); kkayo@sfc.keio.ac.jp (K.K.); 2 Health Science Laboratory, Keio Research Institute at SFC, Kanagawa 252-0882, Japan; t17390qz@sfc.keio.ac.jp; 3 Department of Environment and Information Studies, Keio University, Kanagawa 252-0882, Japan; 4 Department of Ophthalmology, Keio University School of Medicine, Tokyo 160-8582, Japan

**Keywords:** non-alcoholic steatohepatitis, non-alcoholic fatty liver disease, *Agaricus brasiliensis* KA21, anti-oxidant, anti-inflammation

## Abstract

Non-alcoholic steatohepatitis (NASH) is a progressive disease that occurs in the liver. As the number of people with NASH has increased, effective prevention and treatment strategies are needed. *Agaricus brasiliensis* KA21 (AGA) is a mushroom native to Brazil and is considered a healthy food because of its purported health benefits, including its antioxidant properties. In this study, we focused on the oxidative stress that accompanies the onset of NASH and examined whether AGA can prevent NASH development through its antioxidant activity. We used a mouse model of NASH in which pathogenesis was promoted by dietary induction. Supplementation with AGA attenuated the development of hepatic fibrosis, which is a characteristic feature of late-stage NASH. This effect appeared to be mechanistically linked to an AGA-promoted reduction in hepatic oxidative stress. These results demonstrate a novel role for AGA in NASH prevention.

## 1. Introduction

*Agaricus brasiliensis* KA21 (AGA) (or *subrufescens*) is a fungus considered to be a health food because of its antioxidant [1,2] and anti-inflammatory properties [3,4,5]. Inflammation and oxidative stress contribute to lifestyle-related diseases, such as obesity and diabetes. Multiple studies have demonstrated that AGA can ameliorate the symptoms of lifestyle-related diseases [6,7,8], including obesity [9,10], hypertension [11], and diabetes [12,13]. Furthermore, it has been shown that AGA has anti-tumor [14,15], cancer suppression [16,17,18], and immune-enhancing properties [19,20,21,22,23,24,25].

Non-alcoholic steatohepatitis (NASH) is a disease caused by the development of non-alcoholic fatty liver disease (NAFLD) in the liver and the progression of its symptoms [26]. Since 2017, the global prevalence of NAFLD has reached 25.2%, and the prevalence of NASH is 1.5%–6.45% of the adult population [27]. NASH is associated with hepatic fibrosis and increases the risk of developing cirrhosis [28,29] and hepatocellular carcinoma [23,30]. Because fibrosis, a characteristic symptom of NASH, cannot be cured once it has developed, methods of preventing NASH are urgently needed. However, the detailed mechanisms of NASH pathogenesis remain unclear. NASH is a multi-factorial condition characterized by inflammation and oxidative stress, making it challenging to dissect its underlying mechanisms [31,32,33,34]. Acute inflammation is considered part of the complex biological response to resolve injury and neutralize harmful stimuli [35]. NASH is an inflammatory disorder, and nuclear factor-κB and c-Jun N-terminal kinase (JNK) are the two key pro-inflammatory signaling pathways in NASH [36]. Previous studies have shown that inflammation is a key predictor of eventual histological progression to fibrosis and cirrhosis [37]. In agreement with this, substances with anti-inflammatory properties have been used to treat NASH [38,39] Based on this information, we hypothesized that substances with anti-inflammatory properties may have beneficial impacts on NASH.

It has been demonstrated that antioxidants have the potential to function as very effective factors in preventing and treating the onset of NASH pathogenesis [40]. In recent years, antioxidant compounds, such as vitamin E and astaxanthin, have been shown to have preventive and therapeutic effects on NASH [41,42].

Although anti-inflammatory and antioxidant molecules may affect NASH, no cures have been identified.

## 2. Materials and Methods

### 2.1. Materials

The *Agaricus brasiliensis* KA21 strain was obtained from TOEI SHINYAKU Co. Ltd. (Mitaka, Tokyo, Japan). The main composition is shown below (Table 1). The 100 g dry weight was measured by Japan Food Research Laboratories (Shibuya, Tokyo, Japan).

### 2.2. Animal Studies

All animal procedures were performed in accordance with the standards set forth in the Guidelines for the Use and Care of Laboratory Animals at Keio University, Japan. The protocols were approved by the Institute for Experimental Animals of Keio University. Male C57BL/6J mice, five weeks of age (*n* = 21), were obtained from Japan SLC, Inc. (Hamamatsu, Japan). All mice were maintained in a temperature-controlled (23 °C) facility with a 12 h light/dark cycle and were given free access to food and water over a period of 21 weeks, prior to sacrifice. Body weights were recorded regularly, as shown in the results. The mice were divided into three experimental groups (*n* = 7/group). The control group was fed a normal diet. The high-fat and high-cholesterol (HC) group was fed a high-fat and high-cholesterol diet. The third group (AGA) was fed a high-fat and high-cholesterol diet, but with *A. brasiliensis* KA21 added (5% *w/w*). Animal feeds were obtained from Research Diets, Inc., (New Brunswick, NJ, USA). The high-fat and high-cholesterol (HC) diet (D09100301) contained 40 kcal% fat (trans fat 30 kcal%), 40 kcal% carbohydrates (20 kcal% fructose), 20 kcal% protein, and 2% *w*/*w* cholesterol. This diet has been used in several NASH studies to prepare a dietary NASH mouse model [43,44]. The matched control diet (D09100304) contained 10 kcal% fat, 70 kcal% carbohydrates, and 20 kcal% protein. The detailed composition is shown below (Table 2). All mice were fasted for 6 h before harvesting their blood and tissues for analysis, including RNA isolation and histology.

### 2.3. mRNA Expression Analysis by Quantitative RT-PCR

Total RNA was extracted from the tissue samples using the RNeasy Mini Kit (Qiagen, Hilden, Germany). cDNA was synthesized from total RNA with the Prime Script RT Reagent Kit (Takara, Shiga, Japan). Expression levels were analyzed using cDNA synthesized from total mRNA using a real-time PCR cycler. The sequences of the primer sets used are shown in Table 3. The expression levels of all genes were normalized to that of 18S.

### 2.4. Blood Chemistry and Metabolite Analysis

Plasma samples were collected at the time of sacrifice. Plasma was collected from whole blood by centrifugation at 1500 rpm and 4 °C, for 15 min. Plasma total cholesterol triglycerides (TGs) were determined by enzymatic assay kits (Labo Assay^TM^ series, Wako Laboratory Chemicals, Osaka, Japan). Alanine aminotransferase (ALT) and serum aspartate aminotransferase (AST) were determined by the alanine aminotransferase (ALT or SGPT) Activity Colorimetric/Fluorometric Assay Kit (Bio Vision, Milpitas, California, USA).

### 2.5. Liver and Feces Lipid Analysis and Extraction

To measure the liver TGs and cholesterol content, the livers and feces were homogenized in chloroform/methanol (2:1 *v/v*) using a Polytron tissue grinder (Kinematica AG, Luzern, Switzerland). Lipid extracts were prepared by the classical Folch method, as previously described [45]. Extracts were resuspended in isopropanol. 

### 2.6. TBARS Measurement

To measure liver 2-thiobarbituric acid reactive substances (TBARS), the livers were homogenized in tissue protein extraction reagent (T-PER) tissue protein buffer using a Polytron tissue grinder. After centrifugation, the supernatant was collected as a measurement sample using the TBARS Assay Kit (Cayman Chemical Company, Ann Arbor, MI, USA).

### 2.7. Histology and Staining Analysis

Liver tissues were harvested and immediately fixed in 10% neutral buffered formalin (Sigma, St. Louis, MO, USA) and Bouin’s fixative to prepare paraffin-embedded blocks. Hematoxylin and eosin (H&E) staining, Sirius red staining, Masson’s trichrome (MT) staining, and immunohistochemistry were conducted using the paraffin-embedded tissue sections. To quantify the fibrotic area of MT staining, images of eight random fields in each section were processed using ImageJ software (NIH, Bethesda, MD, USA) [46]. The value is shown as a percentage.

### 2.8. Statistical Analysis

Values are reported as the mean ± standard error of the mean (SEM). Statistical differences were determined by analysis of variance (ANOVA), followed by a post-hoc Bonferroni test. Statistical significance is displayed as follows: *p* < 0.05 (^#^), *p* < 0.01 (^##^), and ^#^ significant differences for high-fat and high-cholesterol diet (HC) versus control diet (Control), and *p* < 0.05 (*), *p* < 0.01 (**), and * significant differences for high-fat and high-cholesterol diet (HC) versus high-fat and high-cholesterol + 5% *A. brasiliensis* KA21 (AGA).

## 3. Results

### 3.1. Agaricus Prevents Dietary-Induced NASH

**Figure 1:**
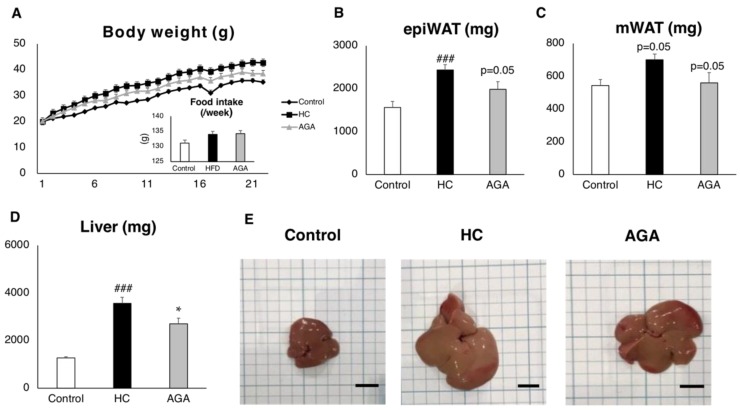
*Agaricus brasiliensis* KA21 (AGA) prevents non-alcoholic steatohepatitis (NASH) onset induced by high-cholesterol (HC) diet. Control diet (Control), HC diet, and high-fat and high-cholesterol +5% AGA. (**A**) Weight change; (**B**) epididymal fat; (**C**) mesenteric fat; (**D**) liver weight; (**E**) liver biopsy pictures’ scale bar shows 10 mm. Data are presented as the mean ± SEM values. *n* = 7 mice per group. Statistical analysis was performed by one-way ANOVA, followed by Bonferroni’s post-hoc test. *p < 0.05; ^###^*p* < 0.001 versus mice in the HC group (^#^ significant differences for HC versus Control, * significant differences for HC versus AGA).

AGA tended to suppress weight gain caused by the HC diet (Figure 1A). Furthermore, the weights of epididymal adipose tissue and mesenteric adipose tissue also tended to be decreased compared to those of the HC only group (Figure 1B,C). In phenotypic analysis of the liver, liver weight was significantly reduced by AGA administration (Figure 1D), and in the biopsy, hypertrophy was suppressed in the AGA administration group compared to in the HC group (Figure 1E).

### 3.2. Agaricus Reduces the Liver Lipids Parameter in HC-Induced Model Mice

**Figure 2:**
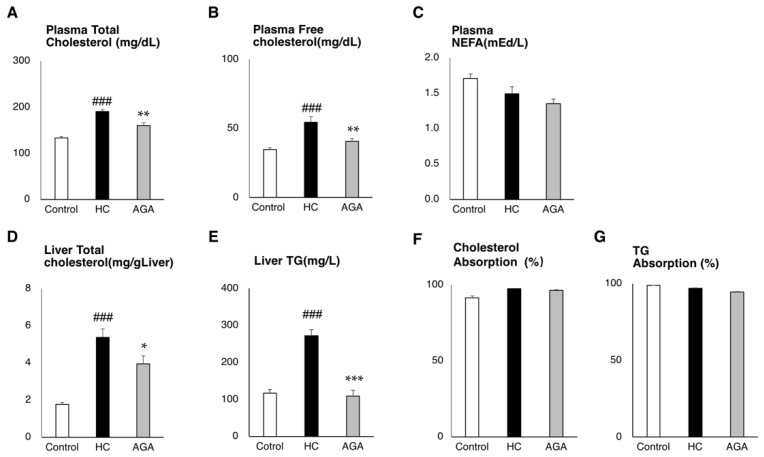
*Agaricus brasiliensis* KA21 (AGA) prevents lipid accumulation. Control diet (Control), high-fat and high-cholesterol diet (HC), and high-fat and high-cholesterol + 5% AGA. (**A**) Plasma lipid parameters for total cholesterol; (**B**) plasma lipid parameters for free cholesterol; (**C**) plasma lipid parameters for non-esterified fatty acid; (**D**) liver lipid parameters for total cholesterol; (**E**) liver lipid parameters for triglyceride (TG); **(F**) percentage of cholesterol absorption; (**G**) percentage of triglyceride (TG) absorption. Data are presented as the mean ± SEM values. *n* = 7 mice per group. Statistical analysis was performed by one-way ANOVA, followed by Bonferroni’s post-hoc test. *p < 0.05; **p < 0.01, ^###^/***p < 0.001 versus mice in the HC group (^#^ significant differences for HC versus Control, * significant differences for HC versus AGA).

In the plasma collected at the time of dissection, the total amount of cholesterol was significantly reduced and was decreased in free-cholesterol and tended to decrease in NEFA (Figure 2A–C). In addition, the measurement of lipids in the liver showed that total cholesterol in the liver was significantly decreased in the AGA group compared to in the HC group (*p*-value = 0.023) and triglyceride (TG) was significantly reduced compared to the HC group (*p*-value < 0.001) (Figure 2D,E). We measured the decrease between the HC and AGA groups. The difference was −1.62 mg/g liver triglyceride, which was a nearly 60% reduction compared to the HC group. The total cholesterol level was −14.07 mg/g liver, showing a 26% decrease in the AGA group compared to the HC group. Cholesterol and triglyceride (TG) absorption rates evaluated from the diet and feces were not significantly different between HC and AGA. This result showed that AGA did not change cholesterol and TG absorption from the diet (Figure 2F,G).

### 3.3. Agaricus Prevents NASH Progression at the Level of Gene Expression and Histological Analysis

**Figure 3:**
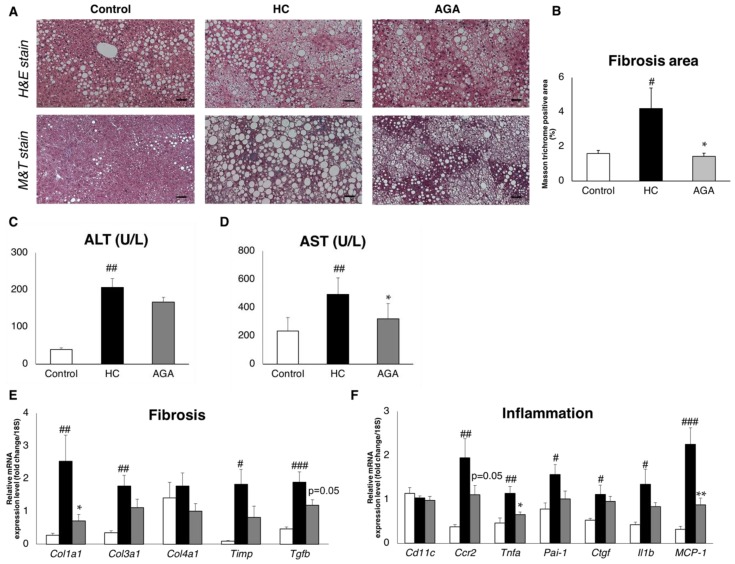
*Agaricus brasiliensis* KA21 (AGA) prevents liver fibrosis and inflammation. Control diet (Control), high-fat and high-cholesterol diet (HC), and high-fat and high-cholesterol +5% AGA. (**A**) Histological staining of the liver (hematoxylin and eosin (H&E) stains’ fat droplets, M&T stains’ collagen fibers), Azan staining, and Sirius red staining; (**B**) evaluation of fibrosis area. Percentage of positive area showing staining within the threshold range to total staining area; (**C**) serum alanine aminotransferase (ALT); (**D**) serum aspartate aminotransferase (AST); (**E**) mRNA expression analysis of fibrosis in the liver (*n* = 7), normalized by using 18S; (**F**) mRNA expression analysis of inflammation in the liver (*n* = 7), normalized by using 18S. Data are presented as the mean ± SEM values. *n* = 7 mice per group. Statistical analysis was performed using one-way ANOVA, followed by Bonferroni’s post-hoc test. ^#^/*p < 0.05; ^##^/**p < 0.01, ^###^*p* < 0.001 versus mice in the HC group (^#^ significant differences for HC versus Control, * significant differences for HC versus AGA).

We performed histological analysis of the liver. While increased lipid droplets were confirmed in the liver of the HC group by H&E staining, the lipid droplets and lipid droplet size were suppressed by AGA administration (Figure 3A). In addition, when masson trichrome stain (M&T) staining was performed, fibrosis was observed in the HC group, whereas less fibrosis was observed in the AGA group (Figure 3A). Quantification of the fibrosis area showed that the fibrosis-positive area was significantly decreased in the AGA group compared to in the HC group (Figure 3B), and in fact returned fibrosis to that of the normal control. We next measured serum alanine aminotransferase (ALT) and serum aspartate aminotransferase (AST). The ALT and AST level were increased in HC group. In the AGA group, both the ALT and AST level decreased, especially the AST level, which was significantly decreased (*p*-value = 0.014) compared to the HC group (Figure 3C,D). This result showed that AGA improved the liver function.

To confirm whether the suppression of liver fibrosis by AGA is regulated at the gene level, we evaluated the expression of collagen type 1 alpha (*Col1a1*) and collagen type 3 alpha 1 (*Col3a1*), as markers of fibrosis transforming growth factor-beta (Tgfb) which are known as upstream regulators of *Col1a1* and *Col3a1*. Expression analysis revealed that these genes were upregulated in the HC group and associated with the onset of NASH. In contrast, in the AGA group, AGA administration was associated with the suppression of these genes. As a result, fibrosis progression in the liver was suppressed at the molecular level (Figure 3E). Moreover, genes such as tumor necrosis factor-alpha (Tnfa) and interleukin 1-beta (Il1b), which are markers of inflammation, were upregulated upon the administration of HC, whereas their expression was reduced upon the administration of AGA (Figure 3F).

### 3.4. Agaricus Reduces Oxidative Stress

**Figure 4:**
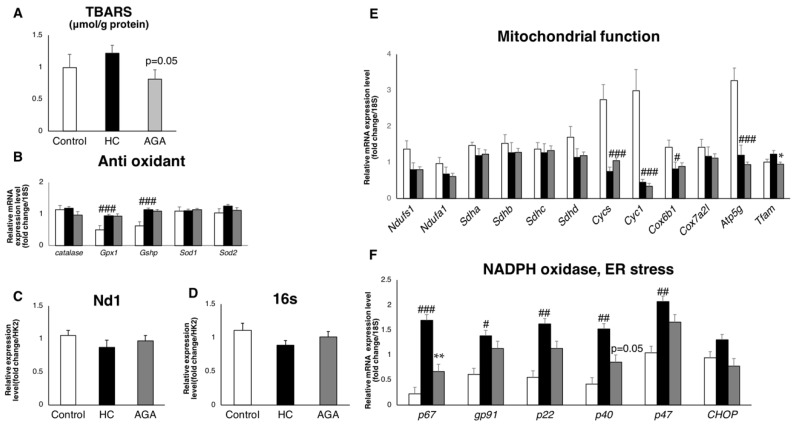
*Agaricus brasiliensis* KA21 (AGA) reduces oxidative stress in the liver. Control diet (Control), high-fat and high-cholesterol diet (HC), and high-fat and high-cholesterol + 5% AGA. (**A**) Lipid peroxide; (**B**) expression of an antioxidant enzyme in the liver; (**C**) mitochondrial copy number (Nd1) in the liver; (**D**) mitochondrial copy number (16S) in the liver; (**E**) gene expression for the mitochondrial complex in the liver (*n* = 7), normalized by using 18S rRNA; (F) gene expression for nicotinamide adenine dinucleotide phosphate (NADPH) (free radical source) activity in the liver (*n* = 7), normalized by using 18S rRNA. Data are presented as the mean ± SEM values. *n* = 7 mice per group. Statistical analysis was performed by one-way ANOVA, followed by Bonferroni’s post-hoc test. ^#^/*p < 0.05; ^##^/**p < 0.01, ^###^*p* < 0.001 versus mice in the HC group (^#^ significant differences for HC versus Control, * significant differences for HC versus AGA).

Next, we focused on the production and elimination systems of reactive oxygen species (ROS), which directly cause oxidative stress. The measurement of typical superoxide dismutase, catalase, and glutathione peroxidase as antioxidant enzymes showed that the expression of Gpx1, which is an enzyme for detoxifying H_2_O_2_ and generated by the electron transfer system, was predominantly elevated in AGA (Figure 4B). We also performed mitochondrial function analysis to measure mitochondrial complexes and mtDNA; however, there were no significant differences between the HC and AGA group (Figure 4C–E). Furthermore, mRNA expression analysis was used to estimate the activity of NADPH oxidase, an enzyme that produces free radicals. The expression of genes that form the NADPH complex tended to be increased in the HC group, whereas several were significantly decreased in the AGA group (Figure 4F). Notably, lipid peroxidation (2-thiobarbituric acid reactive substances (TBARS)), which is a marker of oxidative stress, was reduced in the AGA group (Figure 4A) and again returned the level of oxidative stress to that of the normal control. This result suggests that AGA reduces the oxidative stress that causes NASH, thus preventing disease progression.

**Figure 5:**
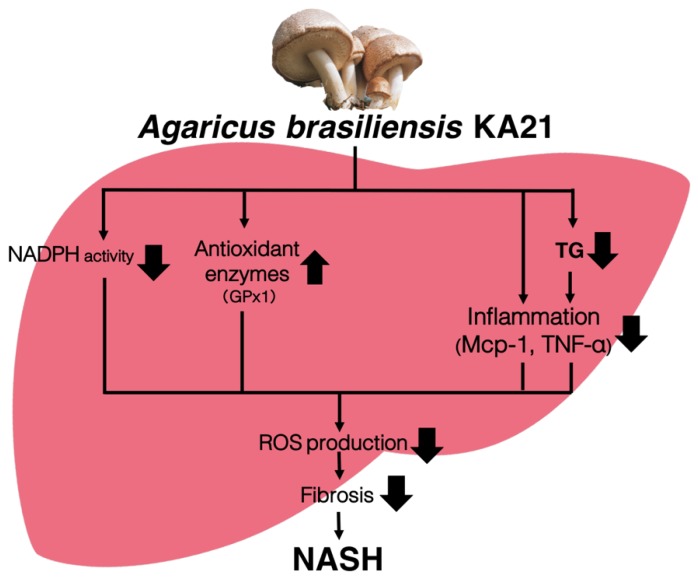
Expected pathway for non-alcoholic steatohepatitis (NASH) prevention by *Agaricus brasiliensis* KA21. NASH; reactive oxygen species (ROS); free fatty acids (FFA); triglycerides (TG); 2-thiobarbituric acid reactive substances (TBARS); nicotinamide adenine dinucleotide phosphate (NADPH); glutathione peroxidase (Gpx1); monocyte chemotactic protein-1 (MCP-1); tumor necrosis factor alpha (Tnfa).

Based on these data, we hypothesized that *A. brasiliensis* KA21 can suppress inflammation/fibrosis in the liver by suppressing the production system of ROS, which is a source of oxidative stress, and promotes their removal (Figure 5).

## 4. Discussion

In this study, we evaluated the impact of AGA administration on oxidative stress and inflammation in diet-induced NASH model mice. Our results suggest that AGA has complex functions in NASH pathologies. Several studies have shown that anti-inflammation is a key factor in the treatment of NASH [39]. One type of polyphenol, resveratrol, ameliorates NASH by decreasing liver fibrosis and inflammation. Resveratrol decreased the fibrosis area by approximately 60% compared to in the NASH model mice. Additionally, inflammation was inhibited and the expression of inflammatory cytokines was decreased. The fibrosis area in the AGA group was decreased by 65% compared to in the HC group. Additionally, decreased levels of inflammatory genes, tumor necrosis factor alpha (Tnfa), and monocyte chemoattractant protein 1 (MCP-1) in the liver suggest the suppression of liver inflammation. These results are consistent with those of a previous study [39]. We hypothesized that AGA has a direct effect on the liver, suppressing inflammation and NASH progression. A previous study showed that linoleic acid isolated from *A. brasiliensis* KA21 inhibits NO production and suppresses the expression of genes encoding pro-inflammatory cytokines, including Tnfa, interleukin 6 (Il6), interleukin 1-beta (Il1b), and nitric oxide synthase 2 (Nos2) in murine leukemia macrophage cell (RAW 264.7 cells) [5]. Our results showed that NO-related genes, particularly *p67^phox^*, *p40^phox^*, and pro-inflammatory cytokines, were decreased in the AGA group. The prevention of inflammation may be mediated by the NO production pathway, as observed previously.

Additionally, characteristic effects of AGA significantly reduced the TG contents and TBARS in the liver. A previous study revealed that astaxisantin, an antioxidant carotenoid, prevented diet-induced NASH by decreasing liver TG contents by approximately 30% and decreasing lipid peroxidation (TBARS) by 30% compared to the high-fat and high cholesterol diet in the liver [43], which was the same diet as used in our experiments. In our data, the liver TG contents were decreased by 59% and TBARS was decreased by 33% compared to in the HC group. This suggests that the improvement resulting from AGA is similar to that of other natural compounds, which may be effective for treating NASH, according to previous studies.

The onset of inflammation and fibrosis in the liver is closely linked to the accumulation of oxidative stress. Oxidative stress is elevated when there is an imbalance between the generation of ROS and antioxidant pathways that remove ROS. ROS accumulation causes damage to cells and tissues by causing DNA damage and lipid peroxidation. TBARS is a marker of oxidative stress because of the excessive activity of NADPH and dysfunction of antioxidant defenses. In our study, AGA reduced TBARS accumulation and the expression of NADPH-related genes.

Our results suggest that AGA is effective for preventing the onset of NASH. The administration of AGA activated anti-inflammatory and antioxidant pathways, which has not been reported previously. Furthermore, AGA administration suppressed NADPH oxidase, the pathway that produces ROS and results in oxidative stress in the liver. Additionally, AGA administration prevented oxidative stress by increasing Gpx1, an enzyme that eliminates ROS. Furthermore, several possible factors, in addition to the suppression of ROS generation, may influence the suppression of hepatic inflammation pathways following AGA administration.

There were some limitations to the study. AGA contains β-glucan, some polyphenols, and vitamins, which are considered bioactive compounds [24], so AGA may not function alone, but rather with multiple putative bioactive compounds. To fully understand the characteristics of AGA, it is also important to evaluate it in further mice studies. A previous study suggested that *A. brasiliensis* KA21 reduces body fat and BMI in healthy humans [24]; however, the detailed mechanism of this is unclear. Further studies are needed to evaluate these effects in humans. When considering the application in humans, it is known that the diet of patients with NASH contains more than two-fold higher levels of fat and carbohydrates compared to that of healthy individuals [47]. Therefore, the NASH diet used in this study reflects the actual diet of patients with NASH.

We found that AGA may regulate and prevent the development of dietary-induced NASH onset at the molecular level. From these findings, AGA may be useful for preventing NASH.

## Figures and Tables

**Table 1:** Composition of *Agaricus brasiliensis* KA21.

Ingredient	/100 g	Ingredient	/100 g
Energy	288.0 kcal	Selenium	88.0 μg
Protein	38.5 g	Arsenicum	0 .5 ppm
Fat	2.6 g	Cadmium	2.0 ppm
Carbohydrate	27.7 g	Plumbum	0.1 ppm
β-glucan	12.4 g	Mercury	0.2 ppm
Fiber	20.6 g	Vitamin B (total caronene)	
Sodium	8.4 mg	Vitamin B1 (Thiamin)	0.6 mg
Calcium	22.5 mg	Vitamin B2 (Riboflavin)	3.0 mg
Iron	10.1 mg	Vitamin B6	0.5 mg
Potassium	2920.0 mg	Niacin	33.5 mg
Phosphorus	952.0 mg	Pantothenic acid	22.9 mg
Magnesium	96.5 mg	Folic acid	230.0 μg
Zinc	7.9 mg	Biotin	123.0 μg
Copper	7.7 mg		
Manganese	0.8 mg		
Vitamin D	56.7 μg		
Agaritine	15.3 ppm		

**Table 2:** Composition of control diet (D09100304) and high-fat and high-cholesterol diet (D09100301).

Product	High-Fat/Cholesterol Diet (D09100301)		Control Diet (D09100304)	
	**gm%**	**kcal%**	**gm%**	**kcal%**
Protein	22.5	20.0	19.2	20.0
Carbohydrate	44.9	40.0	67.3	70.0
Fat	19.9	40.0	4.3	10.0
Total		100.0		100.0
kcal/gm	4.5		3.9	
**Ingredient**	**gm**	**kcal**	**gm**	**kcal**
Casein, 80 Mesh	200.0	800.0	200.0	800.0
L-Cystine	3.0	12.0	3.0	12.0
Corn Starch	0.0	0.0	350.0	1400.0
Maltodextrin 10	100.0	400.0	85.0	340.0
Fructose	200.0	800.0	0.0	0.0
Glucose	0.0	0.0	169.0	676.0
Sucrose	96.0	384.0	96.0	384.0
Cellulose, BW200	50.0	0.0	50.0	0.0
Soybean Oil	25.0	225.0	25.0	225.0
Primex Shortening	135.0	121.0	0.0	0.0
Lard	20.0	180.0	20.0	180.0
Mineral Mix S10026	10.0	0.0	10.0	0.0
DiCalcium Phosphate	13.0	0.0	13.0	0.0
Calcium Carbonate	5.5	0.0	5.5	0.0
Potassium Citrate, 1 H2O	16.5	0.0	16.5	0.0
Vitamin Mix V10001	10.0	40.0	10.0	40.0
Choline Bitartrate	2.0	0.0	2.0	0.0
Cholesterol	18.0	0.0	0.0	0.0
FD&C Yellow Dye #5	0.1	0.0	0.0	0.0
Total	904.1	4056.0	1055.1	4057.0

**Table 3:** Primer sequences.

	Forward Primer (5′→3′)	Reverse Primer (5′→3′)
*16S*	CCGCAAGGGAAAGATGAAAGAC	TCGTTTGGTTTCGGGGTTTC
*18S*	TTCTGGCCAACGGTCTAGACAAC	CCAGTGGTCTTGGTGTGCTGA
*Acc*	ACCCACTCCACTGTTTGTGA	CCTTGGAATTCAGGAGAGGA
*Atp5g*	CACTGCTCATTTCTCCAGCTC	CAGGAAGGCTGCTTAGATGG
*catalase*	CCAGCGACCAGATGAAGCAG	CCACTCTCTCAGGAATCCGC
*Ccr2*	AGCACATGTGGTGAATCCAA	TGCCATCATAAAGGAGCCA
*Cd11c*	AAGAACTGTGGAGCTGACCA	CCACCAGGGTCTTCAAGTCT
*CHOP*	AGGTGAAAGGCAGGGACTCA	CCACCACACCTGAAAGCAGAA
*Col1a1*	CCTCAGGGTATTGCTGGACAAC	TTGATCCAGAAGGACCTTGTTTG
*Col3a1*	TTGATGTGCAGCTGGCATTC	GCCACTGGCCTGATCCATAT
*Col4a1*	CACATTTTCCACAGCCAGAG	GTCTGGCTTCTGCTGCTCTT
*Cox6b1*	ATGTCTCCGTGTGTGAGTGG	GATCTTCCCAGGAAATGTGC
*Cox7a2l*	TTTGGTTGGTGTGGCAAATA	AGTTTCACGCAGAAGTTGGC
*Ctgf*	ACCCGAGTTACCAATGACAATACC	CCGCAGAACTTAGCCCTGTATG
*Cyc1*	GCTTCCAGGTGCAAGTGCT	CAGACTTCGAGGACAAGGACA
*Cycs*	GCAAHCATAAGACTGGACCAAA	TTGTTGGCATCTGTGTAAGAGAATC
*Cyp2e1*	TCTGAGATATGGGCTCCTGA	ATGCACTACAGCGTCCATGT
*Fas*	TCTGCCAGTGAGTTGAGGAC	CTGCAGAGAAGCGAGCATAC
*Gp91*	TTGGGTCAGCACTGGCTCTG	TGGCGGTGTGCAGTGCTATC
*Gpx1*	AGTCCACCGTGTATGCCTTCT	GAGACGCGACATTCTCAATGA
*Gshp*	CCTTGCCAACACCCAGTGA	CCGGAGACCAAATGATGTACTTG
*Il1b*	CTGAACTCAACTGTGAAATGCCA	AAAGGTTTGGAAGCAGCCCT
*MCP-1*	CCACTCACCTGCTGCTACTCAT	TGGTGATCCTCTTGTAGCTCTCC
*Nd1*	CTAGCAGAAACAAACCGGGC	CCGGCTGCGTATTCTACGTT
*Ndufa1*	GTCCACTGCGTACATCCACA	ATCGCGTTCCATCAGATACC
*Ndufs1*	GGAACTACTCGGTGGGCTC	GCCAGTTGTGCGAACATATC
*p22*	GTCCACCATGGAGCGATGTG	CAATGGCCAAGCAGACGGT
*p40*	GCAGGCTCAGGAGGTTCTTC	CGCCGCTATCGCCAGTTCTAC
*p47*	AGATGTTCCCCATTGAGGCCG	GTTTCAGGTCATCAGGCCGC
*p67*	CTGGCTGAGGCCATCAGACT	AGGCCACTGCAGAGTGCTT
*Pai-1*	GCATAGCCAGCACCGAGGA	TCAGCCCTTGCTTGCCTCAT
*Scd-1*	CTCCTGCTGATGTGCTTCAT	AAGGTGCTAACGAACAGGCT
*Sdha*	ATGACACTGTGAAAGGCTCCGACT	TTCCCAAACTTGAGGCTCTGTCCA
*Sdhb*	GCCGTTCTCGGCAGAGTG	TCTGGGTCCCATCGGTAAAT
*Sdhc*	AGTTTGTGCTTGTCTTCCCG	CACTCCAGACAGCCAGACCT
*Sdhd*	CATGGCGGTTCTCTTAAAGC	TGACACATAAGCGGGTCTGA
*Sod1*	TGAGGTCCTGCACTGGTAC	CAAGCGGTGAACCAGTTGTG
*Sod2*	TTAACGCGCAGATCATGCA	GGTGGCGTTGAGATTGTTCA
*Tfam*	ATGTCTCCGGATCGTTTCAC	CCAAAAAGACCTCGTTCAGC
*Tgfb*	TTGCTTCAGCTCCACAGAGA	TGGTTGTAGAGGGCAAGGAC
*Timp*	AGGTGGTCTCGTTGATTTCT	GTAAGGCCTGTAGCTGTGCC
*Tnfa*	CTGGGACAGTGACCTGGACT	GCACCTCAGGGAAGAGTCTG
